# Draft Genome Sequences of Six Type Strains of the Genus *Massilia*

**DOI:** 10.1128/MRA.00226-20

**Published:** 2020-04-30

**Authors:** Henrike Miess, Andri Frediansyah, Markus Göker, Harald Gross

**Affiliations:** a Department of Pharmaceutical Biology, Pharmaceutical Institute, University of Tübingen, Tübingen, Germany; b Research Division for Natural Product Technology (BPTBA), Indonesian Institute of Sciences (LIPI), Wonosari, Indonesia; c Department of Microorganisms, Leibniz Institute DSMZ–German Collection of Microorganisms and Cell Cultures, Braunschweig, Germany; d German Center for Infection Research (DZIF), Tübingen Partner Site, Tübingen, Germany; University of Southern California

## Abstract

High-quality draft genome sequences were determined for 6 *Massilia* sp. type strains. The genomes of these strains show considerable biosynthetic potential for producing secondary metabolites.

## ANNOUNCEMENT

Gram-negative bacteria have become a promising source for novel bioactive natural products (1, 2). As part of our ongoing efforts to investigate natural products from Gram-negative bacteria with significant application in the pharmaceutical-medical or agricultural contexts (3–10), we are currently investigating the genus *Massilia*. Bacteria of this genus are foremost known in a medical context as pathogenic bacteria, causing wound infections, lymphadenopathy, and corneal abscesses (11–13). However, *Massilia* spp. are ubiquitous in the environment since they are associated with plants and can be found in soil and drinking water (14–17). From a chemical point of view, *Massilia* spp. are considered underexplored. To date, solely the production of violacein, *N*-acyl-homoserine-lactones, and the siderophore massiliachelin has been reported (18–20). However, taking into account the empirical relationship between genome size and number of biosynthetic gene clusters (BGCs) encoding secondary metabolites (21, 22) and the average size of the *Massilia* genomes reported so far (ranging from 5.0 to 6.1 Mbp) (23–29), a higher biosynthetic capacity for secondary metabolism can be expected. In order to probe this hypothesis, we initiated the sequencing of six *Massilia* sp. type strains (16, 30, 31; Table 1).

**TABLE 1:** Genome sequence information

Organism^*a*^ (reference)	Genome size (bp)	G+C content (mol%)	Coverage (×)	No. of contigs	No. of reads^*b*^	Subread *N*_50_ (bp)^*c*^	Total CDSs^*d*^	No. of BGCs^*e*^	WGS accession no.	SRA accession no.
*M. albidiflava* DSM 17472^T^ (16)	7,437,157	65.8	129	1	110,512	12,885	6,072	10	CP036401	SRP187148
*M. dura* DSM 17513^T^ (16)	6,913,453	65.6	108	2	116,237	9,787	5,774	6	WNWM00000000	SRP233770
*M. flava* DSM 26639^T^ (30)	6,922,031	66.5	146	1	107,359	13,201	5,880	9	CP046904	SRP238041
*M. lutea* DSM 17473^T^ (16)	7,504,849	64.7	107	1	95,944	11,887	6,209	6	CP035913	SRP185878
*M. plicata* DSM 17505^T^ (16)	5,887,622	65.0	86	1	72,313	10,586	5,094	11	CP038026	SRP190095
*M. umbonata* DSM 26121^T^ (31)	7,426,165	65.3	169	1	135,084	12,829	6,123	9	CP040017	SRP195528

a All type strains were obtained from the German Collection of Microorganisms and Cell Cultures (DSM).

b Total number of reads that passed filtering.

c Subread *N*_50_; 50% of all filtered subreads are longer than the indicated value.

d CDSs, coding sequences.

e BGCs, biosynthetic gene clusters coding for secondary metabolites.

All type strains were obtained from the German Collection of Microorganisms and Cell Cultures (DSMZ). Genomic DNA of the *Massilia* strains was harvested from cultures grown for 2 to 3 days at 27°C in 15 to 20 ml nutrient broth (NB) on a rotary shaker (180 rpm) and isolated as previously described (10). Subsequently, whole-genome sequencing of all strains was performed on a PacBio RS II platform. The libraries were prepared according to the manufacturer’s instructions using a single-molecule real-time (SMRT) cell 8Pac v3 and the P6 DNA polymerase binding kit (PacBio). *De novo* assembly was performed utilizing HGAP3, whose protocol relies on PreAssembler v1 for filtering, PreAssembler v2 and AssembleUnitig v1 for assembly, BLASR v1 (32) for mapping, and Quiver v1 for consensus polishing. All HGAP3 settings were kept at the defaults, except for the genome size estimate parameter, which was set to 8.0 Mbp. Annotation was performed with the NCBI Prokaryotic Genome Annotation Pipeline (PGAP) v4.7 (33, 34).

The sequenced *Massilia* strains shared similar G+C contents (64.7 to 66.5%), which is consistent with the genus description (35, 36), but varied considerably in their genome size (5.9 to 7.5 Mbp) and consequently in the number of coding sequences (Table 1). In the case of M. flava, the genome size was in good agreement with that of an already-reported genome of this species (*M. flava* CGMCC1.10685, GenBank accession no. VLKW00000000) (37); however, otherwise, the *Massilia* strains of this study show a larger size (average 7.0 Mbp) than *Massilia* genomes so far reported in the literature (average, 5.6 Mbp) (23–29). Each of the six strains carried one circular chromosome, and in the strain *M. dura* DSM 17513^T^, one additional extrachromosomal circular replicon, 38.9 kbp in size, was identified by *in silico* prediction. Secondary metabolism analysis using antiSMASH v5.0 (38) with default settings predicted 6 to 11 biosynthetic gene clusters per strain (Table 1), revealing the encouraging potential of this genus toward the production of novel bioactive compounds. The genome sequences will therefore be useful in the search for new natural bioactive products.

### Data availability.

All six whole-genome sequencing (WGS) projects in this study have been deposited at DDBJ/ENA/GenBank, and the corresponding raw sequencing data are available from the Sequence Read Archive (SRA). The accession numbers of interest are provided in Table 1.
